# Longitudinal whole-brain analysis of multi-subject diffusion data in diffuse axonal injury

**DOI:** 10.1590/0004-282X-ANP-2020-0595

**Published:** 2022-03-31

**Authors:** Daphine Centola Grassi, Ana Luiza Zaninotto, Fabrício Stewan Feltrin, Fabíola Bezerra de Carvalho Macruz, Maria Concepción García Otaduy, Claudia da Costa Leite, Vinicius Monteiro de Paula Guirado, Wellingson Silva Paiva, Celi Santos Andrade

**Affiliations:** 1 Universidade de São Paulo, Faculdade de Medicina, Hospital das Clínicas, Departamento de Radiologia, São Paulo SP, Brazil. Universidade de São Paulo Faculdade de Medicina Hospital das Clínicas São Paulo SP Brazil; 2 Universidade de São Paulo, Faculdade de Medicina, Hospital das Clínicas, Laboratório de Investigação Médica 44, São Paulo SP, Brazil. Universidade de São Paulo Faculdade de Medicina Hospital das Clínicas São Paulo SP Brazil; 3 Massachusetts General Hospital, Institute of Health Professions, Speech and Feeding Disorders Lab, Boston, USA. Massachusetts General Hospital Institute of Health Professions Speech and Feeding Disorders Lab Boston USA; 4 Universidade de São Paulo, Faculdade de Medicina, Hospital das Clínicas, Departamento de Neurologia, São Paulo SP, Brazil. Universidade de São Paulo Faculdade de Medicina Hospital das Clínicas São Paulo SP Brazil; 5 University of Texas Southwestern Medical Center, Dallas, USA. University of Texas Southwestern Medical Center Dallas USA; 6 Alliar Group, São Paulo SP, Brazil. Alliar Group São Paulo SP Brazil

**Keywords:** Craniocerebral Trauma, Diffuse Axonal Injury, Diffusion Tensor Imaging, Glasgow Outcome Scale, Regeneration, Traumatismos Craniocerebrais, Lesão Axonal Difusa, Imagem de Tensor de Difusão, Escala de Resultado de Glasgow, Regeneração

## Abstract

**Background:**

Diffuse axonal injury occurs with high acceleration and deceleration forces in traumatic brain injury (TBI). This lesion leads to disarrangement of the neuronal network, which can result in some degree of deficiency. The Extended Glasgow Outcome Scale (GOS-E) is the primary outcome instrument for the evaluation of TBI victims. Diffusion tensor imaging (DTI) assesses white matter (WM) microstructure based on the displacement distribution of water molecules.

**Objective:**

To investigate WM microstructure within the first year after TBI using DTI, the patient’s clinical outcomes, and associations.

**Methods:**

We scanned 20 moderate and severe TBI victims at 2 months and 1 year after the event. Imaging processing was done with the FMRIB software library; we used the tract-based spatial statistics software yielding fractional anisotropy (FA), mean diffusivity (MD), axial diffusivity (AD), and radial diffusivity (RD) for statistical analyses. We computed the average difference between the two measures across subjects and performed a one-sample t-test and threshold-free cluster enhancement, using a corrected p-value < 0.05. Clinical outcomes were evaluated with the GOS-E. We tested for associations between outcome measures and significant mean FA clusters.

**Results:**

Significant clusters of altered FA were identified anatomically using the JHU WM atlas. We found increasing spotted areas of FA with time in the right brain hemisphere and left cerebellum. Extensive regions of increased MD, RD, and AD were observed. Patients presented an excellent overall recovery.

**Conclusions:**

There were no associations between FA and outcome scores, but we cannot exclude the existence of a small to moderate association.

## RESUMO

**Antecedentes:**

A lesão axonial difusa ocorre em traumas com alta energia de aceleração e desaceleração, determinando desorganização da microestrutura cerebral, levando a algum déficit. A escala de Glasgow Estendida (GOS-E) é indicada na avaliação clínica das vítimas de trauma cranioencefalico. Imagens por tensor de difusão (DTI) estudam a microestrutura cerebral a partir da difusão das moléculas de água.

**Objetivo:**

Investigar a microestrutura cerebral no primeiro ano após trauma, avaliar clinicamente os pacientes e testar para correlações entre estes resultados.

**Métodos:**

20 vítimas de TCE moderado e grave foram avaliados 2 meses e 1 ano depois do trauma. O processamento foi feito usando o software FMRIB (FSL) e a análise estatística foi feita com *tract-based spatial statistics software* para extrair os parâmetros de DTI. Calculamos a diferença da média entre as duas observações de cada sujeito e fizemos um teste-t para uma amostra e *threshold-free cluster enhancement*. ﻿Realizamos correções para múltiplas comparações e determinamos o valor de p < 0.05 como significativo. Um ano após o trauma, a avaliação clínica foi feita usando a GOS-E. Testamos para associações entre os resultados clínicos e os valores médios de FA dos clusters.

**Resultados:**

Os clusters significativos foram identificados usando o atlas JHU WM. Observamos aumento de FA predominantemente no hemisfério cerebral direito e cerebelar à esquerda e também extensas áreas de aumento nos demais parâmetros de DTI. A recuperação dos pacientes foi satisfatória.

**Conclusões:**

Não encontramos associações entre os resultados, no entanto alguma associação pequena a moderada não pode ser excluída.

## INTRODUCTION

Traumatic brain injury (TBI) causes different complex brain lesions such as hematomas, contusions, vascular injuries, and diffuse axonal injury (DAI). DAI results from high-energy acceleration and deceleration forces, determining shearing strains in the white matter, leading to disconnection or dysfunction of the neural network^1^.

 Head injuries, particularly DAI, result in distinct functional deficits, such as physical, cognitive, and behavioral impairments, which dramatically affect life quality, return to daily activities, and social reintegration of survivors^2^. In 1975, Jennett and Bond developed the Glasgow Outcome Scale (GOS), and it was used as a primary outcome measure in phase III trials in TBI^3^^,^^4^. Afterward, acknowledging some limitations of the GOS, the Glasgow Outcome Scale -Extended (GOS-E) was developed. Since its establishment in 1981, it has been used and recommended as the primary outcome measurement in TBI studies^5^^,^^6^. 

DAI is not only restricted to mechanical forces at the moment of the trauma. Many different processes are triggered, such as inflammatory responses, molecular changes, apoptosis, and Wallerian degeneration. Therefore, the pathophysiology of DAI can be divided into primary and secondary lesions. The primary axonal lesion is the complete disconnection related to the kinetic energy in the moment of trauma. In contrast, secondary axonal injuries are indirect and progressive lesions in neurons that occur late after the initial shock^7^. The impact sparks molecular and cellular events that disturb the homeostasis, leading to changes in neurons and to the regional microglia that can persist for years^8^. 

 Traditional imaging modalities such as computed tomography and standard magnetic resonance (MR) sequences, such as T1 and T2 weighted sequences, are not sensible enough to show the white matter (WM) damage related to DAI. Diffusion tensor imaging (DTI) is an advanced MR modality based on water molecules diffusion that measures the preferential displacement along the white matter tracts and has been used to assess the brain microstructure in different pathologies, including head injuries^9^. There are diverse methods available to analyze DTI images, such as region-of-interest analysis and tractography. One of the most commonly used is the whole-brain approach for group comparisons, for which tract-based spatial statistics (TBSS) is particularly recommended for voxel wise and cluster-based analyses, constraining statistical analysis to the center of the tracts^9^. It is a semi-automated method, with minimal user-dependence, that allows a whole-brain evaluation and is notably suitable for evaluating diffuse lesions in the brain parenchyma such as DAI^10^^,^^11^. 

 Other groups have used this approach to assess white matter changes in victims of head injury in different stages after trauma^12^^,^^13^. Lipton and colleagues conducted a study on patients with mild TBI who presented with persistent cognitive impairment eight months to three years after the trauma. They found decreased fractional anisotropy (FA) and increased mean diffusivity (MD) in the corpus callosum, subcortical white matter, and internal capsules compared to healthy controls^13^. Another group investigated adolescents with mild TBI in the acute phase (from 1 to 6 days after the trauma event) compared to age-matched controls^14^. They found significantly decreased apparent diffusion coefficient (ADC) and radial diffusivity (RD) and increased FA in several white matter regions and the left thalamus, consistent with axonal cytotoxic edema in the acute phase post-injury. However, few published works analyzed the progressive changes in the white matter in DAI, particularly in moderate and severe trauma victims. 

This study aimed to investigate longitudinally the white matter of patients with severe and moderate DAI at two moments defined as the subacute (two months) and early chronic phases (one year) following the trauma event. We also assessed patients’ clinical outcome one year after trauma using the GOS-E scale^6^. Our central hypothesis is that DTI parameters change with time and can have a degree of correlation with functional outcome.

## METHODS

### Standard protocol approvals

The protocol was reviewed and approved by the institutional review board, the local ethics committee, and all participants gave written informed consent.

### Study design and subjects

A prospective study was conducted throughout one year. Adult outpatients admitted at the Emergency Room of Hospital das Clínicas, Faculdade de Medicina da Universidade de São Paulo, Brazil, victims of moderate and severe TBI (Glasgow Coma Scale scores between 3 and 12 at initial evaluation), presenting clinical and tomographic findings exclusively of DAI were eligible to be included in the study. Exclusion criteria were the presence of contusions greater than 10 cm^3^, midline shift greater than 0.5 cm, extra-axial collection determining compression of the brain parenchyma, or any indication for surgical intervention. Patients with poor quality imaging studies that limited analysis, clinical contra-indications that precluded MR scanning, or loss of follow-up were also excluded.

### Data acquisition 

All data were acquired on a 3T system (Intera Achieva, Philips Healthcare, Best, The Netherlands). Patients were scanned using an 8-channel head proton coil (Philips Healthcare, Best, The Netherlands) at two time-points: two months (subacute phase) and one year (early chronic phase) after the trauma. The routine protocol included fluid-attenuated inversion recovery (FLAIR), diffusion-weighted imaging (DWI), and susceptibility-weighted imaging (SWI) sequences. For the data analysis in this study, we used a volumetric T1-weighted and DTI sequences.

The 3D-T1 fast field echo, acquired in the sagittal plane, was obtained using the following parameters: FOV 240 x 240 x 180 mm^3^; matrix 240 x 240 mm; isotropic resolution; TR/TE 6.2/2.7 ms; and acquisition time 4.13 min.

The DTI sequence was acquired in the axial plane, using 32 directions and one b0 using the following parameters: 70 slices; slice thickness 2 mm; no gap; field of view 256 x 256 mm; voxel resolution = 2 mm^3^ (isotropic); TR/TE 8.500/61 ms; *b* = 1000 s/mm^2^; matrix 128 x 128; number of excitations (NEX) = 1; and acquisition time of 7 minutes.

### Imaging processing and analysis

Initially, all diffusion images were pre-processed for eddy current corrections and extraction of non-brain voxels, using FMRIB's Diffusion Toolbox (FSL) software, version 5.0.11^9^^,^^15^. For motion correction, the free toolbox Explore DTI (A. Leemans, University Medical Center, Utrecht, The Netherlands) was used, which rotates the B-matrix while keeping the exact initial orientation. With this same software, visual quality inspection for residuals and outliers was performed in each data set^16^^,^^17^.

Thereafter, FA maps were analyzed using TBSS^9^. All individual FA images were non-linearly registered to the most typical subject of the sample (using -n command), and then the aligned dataset was transformed into the MNI152 standard space (1 mm^3^). The mean aligned FA images were merged into a single four-dimensional (4D) average FA image. A mean FA skeleton was extracted from the generalized 4D image, and the tracts were projected into the skeleton, using a 0.2 threshold^18^. To extract mean, axial, and radial diffusivities (MD, AD, and RD, respectively), non-linear warps and skeleton projections were applied to each DTI scalar parameter. 

### Statistical analysis

To assess differences in FA, MD, RD, and AD with time, we performed one-sample t-tests, using the average difference between the two measures across subjects. Initially, the difference between the subacute and the early chronic phase was calculated, and then the early chronic value minus the subacute phase value was calculated. Permutation-based nonparametric inferences were made on unsmoothed statistical maps, using 5000 permutations, and the cluster-like structures were enhanced using the threshold-free cluster enhancement (TFCE) algorithm^19^. This approach was similarly applied to the MD, AD, and RD maps. Data were corrected for multiple comparisons, using the family-wise error (FWE) rate, setting the significance level at p < 0.05. 

Thenceforth, the cluster tool (http://fsl.fmrib.ox.ac.uk/fsl/fslwiki/Cluster) was applied to extract the exact clusters, followed by the Atlasquery tool to obtain the coordinates (http://fsl.fmrib.ox.ac.uk/fsl/fslwiki/Atlasquery) according to the Johns Hopkins University (JHU) white matter tractography atlas. 

### Outcome measure

We used the GOS-E at 12 months post-injury obtained at the medical appointment follow-up, which has been recommended as the main outcome measurement in TBI studies^7^. It consists in an eight-scale global measure of function, used to estimate physical disability grading^6^. It classifies patients into upper and lower levels of good recovery (GOS-E = 7 and 8), moderate disability (GOS-E = 5 and 6), severe disability (GOS-E = 3 and 4), vegetative state (GOS-E =2) and death (GOS-E =1). 

### Association analysis 

The WM areas with FA differences with time were defined as ROIs and the mean FA values of each one was calculated. Then, to test for association of mean FA values of each ROI with GOS-E grading, we used Cohen’s d effect size test. We segmented patients into two different groups: sub-optimal (GOS-E= 5 or 6) and optimal (GOS-E = 7 or 8) performance. We tested for associations of each ROI at two months and one year after trauma. 

Taking into account the relatively small patient sample, we also estimated Cohen’s d effect size test considering a bigger sample size (4 times our sample, with the same distribution).

## RESULTS

In the initial screening, 225 patients with head trauma were evaluated, and the final analysis included twenty of those patients. Demographics of the final sample are described in Table 1. Two hundred and five subjects were excluded for the following reasons:

-186 had no clinical and/or tomographic criteria for DAI;
-7 follow-up losses;
-5 were not eligible for MRI;
-5 had low-quality DTI studies;
-1 developed epidural compressive hematoma;
-1 died.

**Table 1.** Demographics of the 20 patients included in the study.

Demographics	
Sex	Male = 16
	Female = 4
Handedness	Right-handed = 16
	Left-handed = 4
Traumatic event	Motorcycle = 10
	Car accident = 6
	Run over = 3
	Agression = 1
GCS at hospital admission	Moderate (GCS 9-12) = 14
	Severe (GCS < 8) = 6
Interval between trauma and hospital admission	39 minutes (15 to 77 minutes)
One-year outcome	GOS-E 5 = 1
	GOS-E 6 = 7
	GOS-E 7 = 11
	GOS-E 8 = 1

GCS: Glasgow coma scale; GOS-E: Glasgow outcome scale extended.

**Figure 1.**
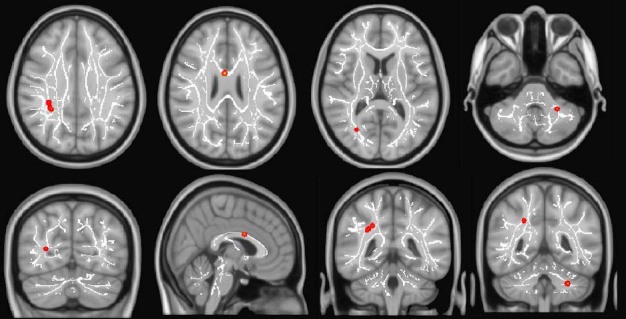
The most significant clusters found with increments in FA (early chronic phase minus subacute phase) are shown in red, TFCE (p < 0.05, FWE corrected). The mean FA skeleton is indicated in white.

**Figure 2.**
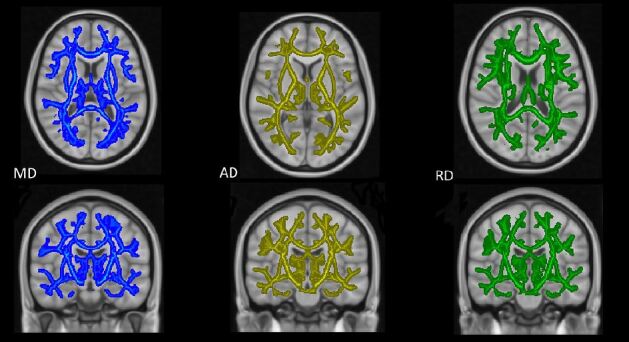
White matter differences between early chronic and subacute phases. Significant clusters (p < 0.05, FWE corrected). Blue depicts MD, yellow AD, and green RD increases in the chronic phase.

FWE: family-wise error; MD: mean diffusivity; AD: axial diffusivity; RD: radial diffusivity.

Evaluation of changes between two months and one year after trauma (chronic minus subacute volumes) with voxel-based TFCE analysis indicated brain regions with FA increment with time, predominantly in the right hemisphere and in the left cerebellum. Significant brain clusters (Table 2) were found in the right superior longitudinal fascicle, the temporal part of the right superior longitudinal fascicle, right inferior fronto-occipital fascicle, right superior and inferior longitudinal fascicles, the body of corpus callosum, forceps major and left corticospinal tract (Figure 1). Moreover, we found extensive areas of increases in MD, RD, and AD (p < 0.05, FWE corrected) (Figure 2). 

**Table 2.** Significant clusters found in FA analysis.

Cluster Index	Voxels	p	X (mm)	Y (mm)	Z (mm)	Location
7	7	0.007	25	-44	36	R SLF, R IFOF
6	4	0.025	3	3	26	body corpus callosum
5	4	0.024	32	-41	30	R SLF, R SLF (temporal part), R ILF
4	2	0.027	-28	-47	-38	L CST
3	1	0.04	29	-68	11	forceps major, R IFOF, R ILF
2	1	0.038	30	-43	32	R SLF and R ILF
1	1	0.031	33	-35	32	R SLF, R SLF (temporal part)

R SLF: right superior longitudinal fascicle; R IFOF: right inferior frontal-occipital fascicle; R ILF: right inferior longitudinal fascicle; L CST: left cortical spinal tract .

FA: fractional anisotropy.

Of note, the one-sample t-test used to assess the difference between subacute and early chronic volumes did not demonstrate significant differences for any DTI parameter.

**Figure 3.**
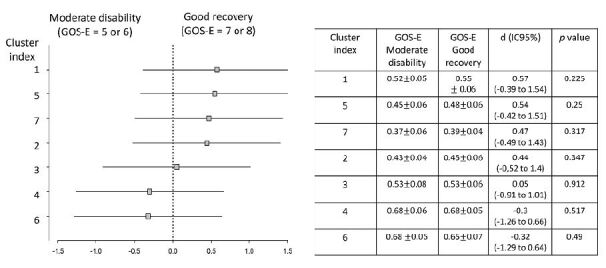
Subgroup analysis. The forest plot shows the effect size in the outcome variable across the pre-specified subgroups according to GOS-E outcome stratification (moderate disability *vs* good recovery). Association analysis between different ROIs at 2 months after trauma with sub-optimal and optimal 1-year post-trauma GOS-E scores. Horizontal axis indicates differences between the groups of recovery according to each cluster. Effect size values are displayed with respective 95% confidence intervals and statistical significance (*p*) obtained by Cohen’s d test (squares).

**Figure 4.**
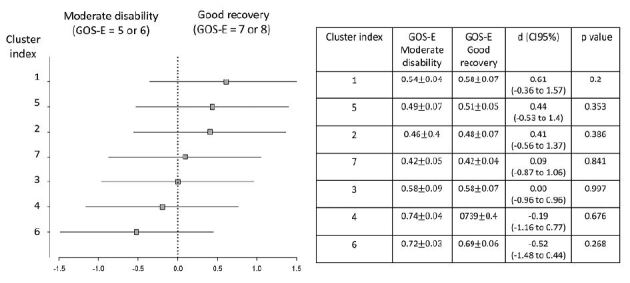
Subgroup analysis. The forest plot shows the effect size in the outcome variable across the pre-specified subgroups according to GOS-E outcome stratification (moderate disability *vs* good recovery). Association analysis between different ROIs at one year after trauma with sub-optimal and optimal 1-year post-trauma GOS-E scores. Horizontal axis indicates differences between groups of recovery according to each cluster. Effect size values are shown with respective 95% confidence intervals and statical significance (*p*) obtained by Cohen’s d test.

Correlations between the different FA ROIs and the one-year GOS-E grades were tested with different ROIs at 2 months and 1 year post-trauma (Figures 3 and 4). We did not find any correlations on either moment.

In addition, by hypothetically increasing our sample 4-fold, we found some associations between one-year GOS-E and the specific ROIs of FA increase at 2 months and 1 year after trauma (Table 3). 

**Table 3.** Correlation analysis artificially increasing the sample size 4-fold.

Cluster index	p value (2 months)	p value (1 year)
1	0.014	0.009
2	0.056	0.078
3	0.823	0.994
4	0.190	0.398
5	0.020	0.061
6	0.161	0.024
7	0.044	0.685

p value obtained by Cohen’s d test.

## DISCUSSION

 In our investigation, we performed whole-brain analysis using a semi-automated method to explore white matter changes over time in moderate and severe TBI victims. DTI has mainly been used to study white matter in the trauma scenario. However, most published articles are related to mild trauma and with different follow-up periods^12^^,^^13^^,^^20^. It is important to emphasize that our patient sample is very homogeneous, consisting of victims with moderate and severe trauma, who were explicitly and exclusively diagnosed with DAI, and followed for one year after the event.

 We found some scattered areas of FA increase, notably in the right brain hemisphere, accompanied by vast regions in the brain and the cerebellum demonstrating an increase in MD, RD, and AD over time. Interestingly, patients showed relatively good clinical outcomes, according to the GOS-E scale. We also found different associations between each brain region with increased FA and the late clinical outcome (GOS-E) two months and one year after trauma, which were more prominent when tested in a larger sample size. Our results are aligned with previous studies that have described white matter changes on DTI parameters over with time in victims of head trauma^21^^,^^22^. These ongoing DTI parameters are related to different pathophysiological processes such as inflammation, degeneration, and regeneration -which have already been described in experimental studies^23^^,^^24^.

 We identified a general area of increase in MD, AD, and RD in brain tracts one year after trauma. We consider that the MD increase is mainly a result of high RD values and, in a lower degree, to AD increment. MD represents the overall diffusivity of water molecules, which can be related to the increasing content of isotropic tissue with water content (gliosis)^25^. Although the biological basis for anisotropy and diffusivity changes in tissues revealed by DTI data is still largely debated, studies using animal models have demonstrated that axonal injury itself is represented by AD changes, and demyelination is associated with an increase in RD values^26^. Considering that increases in both AD and RD contribute positively to increase in MD values, it is reasonable to assume MD as a more sensitive parameter when compared to FA in our observation.

Moreover, in addition to axonal injury, other important and specific pathophysiological processes are also present in the trauma scenario, such as neuroinflammation, afferent degeneration, and debris clearance, and the magnitude of each one at different stages may imply distinct changes in DTI scalar values. Animal model studies play an essential role in characterizing these other effects of the trauma event and how they change over time. However, most of the articles published to date describe the changes that occur in the early acute time after trauma, and only a limited number of articles evaluate long-term consequences^27^. It is already well established that the overall axonal injury in trauma survivors is a consequence of the secondary axonal injury, which is the indirect damage to neurons related to neuroinflammation and microglial activation, triggered by the initial impact and that can persist for years^23^. These processes are responsible for biochemical changes leading to local edema and changes in the microvascular circulation, leading to ischemia and demyelination, which can be confirmed by the RD increase over time^28^. Moreover, AD increase has been associated with an increase of the extracellular water content, such as debris clearance, that would ease the water molecule movement in an axis parallel to the axons^29^. Thereby, we suppose that our results can be explained by the Wallerian or Wallerian-like degeneration process due to DAI or related to a secondary pathological process, such as regional ischemia, and neuronal death may ultimately lead to brain atrophy^29^.

 We also found some spotted areas of FA increase in the right brain hemisphere and the left cerebellum over time. Different causes can be associated with FA increase, such as local fibrosis, hemorrhage areas, and neuronal sprouting^30^. FA is related to the microstructural organization, with high values (close to one) related to most anisotropic tissues. Microstructural organization after trauma has been reported to start in the first few days and can persist for years, which is linked to neuroplasticity^31^. The functional recovery accompanied by the increase in FA may somehow be related to neuroplasticity. Interestingly, we found areas of FA increase in the right brain hemisphere and in the left cerebellum, which may indicate the involvement of the contralateral cerebellar hemisphere in functional and compensatory changes after trauma, as it has been already reported^32^. An interesting functional study compared children with moderate and severe trauma to controls, showing that children with TBI demonstrated changes in functional cerebral activity and increased recruitment of neural resources such as the cerebellum^32^. 

We tested for correlations between mean FA values at the subacute and early chronic phases of the specific regions that presented significant changes over time and the GOS-E scores. We could not find any significant correlations, but the lack of significance may be related to our sample size, which was relatively small when considering the optimal number of individuals required for correlational studies^33^. Still, some specific regions, such as the right SLF and the body of the corpus callosum, demonstrated promising effect sizes in functional stratification at the early chronic phase between optimal and sub-optimal GOS-E scores and mean FA values by using a theoretical larger sample size.

 Whole-brain voxel-wise analysis has been increasingly used to study DAI because of the widespread nature of the disorder and the advantage of this method being minimally invasive for multi-subject group evaluation. However, with this technique, it is imperative the use rigorous statistical procedures to correct for multiple comparison errors, which reduce the sensitivity for detecting subtle changes^12^. 

One limitation of our study is the relatively small sample size. However, we included an homogeneous group of patients with a minimum one-year survival after the traumatic event, especially considering that victims of moderate and severe head trauma have high mortality rates in the first six months^34^^,^^35^. Moreover, these patients also presented an excellent recovery with high one-year GOS-E scores. This may be related to the exclusion of other conditions commonly associated with a head injury, such as contusions and hematomas that are related to a worse outcome^2^. 

 Concerning the methodology and image acquisition, we must emphasize that more gradient encoding directions and more robust DTI acquisition and analytical methods such as high angular resolution diffusion imaging (HARDI), diffusion kurtosis imaging (DKI), and q-ball imaging are available and could have enhanced the power of data analysis^11^^,^^36^. However, these approaches require longer acquisition times, more sophisticated algorithms, and are still not feasible to implement in clinical and research scenarios. 

 In conclusion, our work indicated changes in all DTI scalar metrics in the brain and cerebellum white matter in a homogeneous group of DAI victims along the first year following moderate and severe head trauma. This study can be important to guide future research in understanding the different pathophysiological processes that occur at different stages of patient recovery. Further studies are expected to show that DTI is a tool for signaling functional outcomes and is a promising method to guide therapies and rehabilitation procedures in trauma survivors.
